# Targeted STING Activation Using Modified Ultrasound‐Responsive Microbubbles Enhances Immune Checkpoint Blockade Against Melanoma

**DOI:** 10.1002/advs.202416596

**Published:** 2025-03-26

**Authors:** Sina Khorsandi, Kristin Huntoon, Yifan Wang, Adam Woodward, Abin Antony, Connor Endsley, Nazia Hafeez, Jared L. Edwards, Nicole McCuen, Prasanna G. Alluri, Betty Y.S. Kim, Wen Jiang, Jacques Lux

**Affiliations:** ^1^ Department of Radiology University of Texas Southwestern Medical Center Dallas TX 75390 USA; ^2^ Department of Biomedical Engineering University of Texas Southwestern Medical Center Dallas TX 75390 USA; ^3^ Department of Radiation Oncology MD Anderson Cancer Center Houston TX 77030 USA; ^4^ Department of Neurosurgery MD Anderson Cancer Center Houston TX 77030 USA; ^5^ Department of Radiation Oncology University of Texas Southwestern Medical Center Dallas TX 75390 USA

**Keywords:** cancer therapy, drug delivery, immunotherapy, microbubbles, sting pathway, ultrasound

## Abstract

Despite the recent successes of immune checkpoint inhibitors (ICIs) in treating advanced melanoma, durable clinical responses still remain limited. To boost immune responses, agents that target immune regulators, such as the Stimulator of Interferon Genes (STING) agonist cyclic GMP‐AMP (cGAMP), are being investigated. However, their clinical translation is impeded by poor serum stability, rapid tissue clearance, and T‐cell death due to off‐target activation. Recently, a novel strategy termed Microbubble‐assisted UltraSound‐guided Immunotherapy of Cancer (MUSIC) has been reported to selectively deliver cGAMP directly into the cytosol of antigen‐presenting cells with spatiotemporal control. The resulting activation of STING and downstream proinflammatory pathways produces antitumor effects in murine models of breast cancer. Herein, this study reports that MUSIC provides curative results in aggressive murine models of melanoma as well. Under ultrasound exposure, MUSIC reduces tumor size, increases overall survival, and synergizes with ICIs to bridge innate and adaptive immunities. The results from this study represent MUSIC's ability to produce potent immune responses in melanoma, thus indicating its potential as an adjuvant for cancers where ICI is the current standard of care.

## Graphical abstract

This study explores the use of ultrasound contrast agents loaded with Stimulator of Interferon Genes (STING) agonists as an approach to activate the body's immune system against cancer. Termed Microbubble‐assisted UltraSound‐guided Immunotherapy of Cancer (MUSIC), this strategy effectively inhibits tumor growth and improves survival in mouse models of melanoma, especially when combined with already clinically approved immune checkpoint inhibitors.

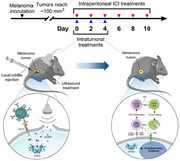

## 1 Introduction

The clinical successes of immune checkpoint inhibitors (ICIs) have ushered in a renaissance era for immunotherapy, as ICIs are now the standard‐of‐care for many cancer types.^[^ ^1^ ^]^ However, patient response to ICIs remains highly heterogeneous, with the majority of patients showing little to no response at all.^[^ ^2^ ^]^ A potential reason for the low efficacy seen in many patients is from a lack of innate immune activation. Certain innate immune cells, such as antigen presenting cells (APCs), possess phagocytic and antigen presenting capabilities that enable the initial immune recognition of tumor cells.^[^ ^3^ ^]^ By presenting tumor‐associated antigens, APCs prime T cells and bridge the innate and adaptive immune systems.^[^ ^4^ ^]^ Unfortunately, many cancers have a “cold” immunosuppressive tumor microenvironment, which prevents the innate immune system from initiating tumor recognition and generating an adequate immune response.^[^ ^5^, ^6^, ^7^ ^]^ Given that ICIs act primarily on the adaptive immune system, a suboptimal innate immune response could lead to failure in ICI therapy.^[^ ^8^ ^]^ Therefore, future strategies need to activate both the innate and adaptive immune systems in order to generate optimal antitumor immunity.^[^ ^9^ ^]^

Recently, the innate immune sensing cyclic GMP–AMP synthase–stimulator of interferon genes (cGAS‐STING) pathway has emerged as a potential therapeutic target to boost antitumor immune responses. Activation of STING promotes transcription of inflammatory cytokines such as type I interferons that are essential for the maturation of APCs and their ability to cross‐present tumor‐derived antigens and upregulate co‐stimulatory molecules.^[^ ^10^, ^11^ ^]^ Interferons are also essential for the complete priming of T cells, and without them, T cell expansion is massively curtailed.^[^ ^12^ ^]^ This indicates that even though cGAS‐STING is an innate immune pathway, it appears to have effects on adaptive immunity as well. Cyclic dinucleotide (CDN) STING agonists such as the natural activator cGAMP are currently being investigated as a potential treatment for a wide range of tumors.^[^ ^13^ ^]^ However, as a cytosolic sensor, STING requires CDNs to penetrate through the cell membrane for its activation. This is highly challenging because the inherent dual negative charges on CDNs repel them from the negatively charged cell membrane. Furthermore, non‐specific STING activation leads to dose‐dependent T cell death and proliferation of regulatory B cells that facilitate tumor immune evasion, further hindering the translation of STING agonists into the clinic.^[^ ^14^, ^15^ ^]^ Therefore, achieving efficient cytosolic delivery of cGAMP into APCs in a controlled and targeted manner would greatly overcome the current limitations and significantly advance the development of STING agonists as a new class of cancer immunotherapeutics.

Previously, we have developed a novel Microbubble‐assisted UltraSound‐guided Immunotherapy of Cancer (MUSIC) strategy consisting of nanocomplex‐conjugated microbubbles (ncMBs) that are targeted to APCs and loaded with cGAMP.^[^ ^16^ ^]^ Upon exposure to ultrasound (US), MUSIC delivered cGAMP into the APC cytosol via sonoporation, resulting in activation of STING and downstream inflammatory pathways. The dual‐specificity mechanisms provided by US and antibody‐mediated targeting enabled spatiotemporal control over activation and reduced off‐target effects. Furthermore, the downstream proinflammatory pathways efficiently primed antigen‐specific T cells and bridged innate and adaptive immunities. Additionally, MUSIC combined synergistically with ICIs, thus showing its potential to increase the efficacy of clinically approved therapeutics. This resulted in MUSIC inhibiting tumor growth and increasing survival time in both localized and metastatic murine breast cancer models. To expand the use of MUSIC to cancers in which ICIs are the first‐line therapy, we investigated the efficacy of MUSIC against melanoma. As ICIs, such as anti‐CTLA‐4 (aCTLA4) and anti‐PD‐1 (aPD1) antibodies, have shown inconsistent curative results in the treatment of melanoma, the use of MUSIC as an adjuvant to ICIs could improve the current standard of care.^[^ ^17^ ^]^ In combination with aCTLA4 and aPD1, MUSIC was able to provide curative effects in two murine melanoma models, showing great synergy with these clinically approved ICIs. Quantification of the immune landscape after treatment also showed increased cytotoxic T lymphocyte and CD11b^+^ immune cell activity, indicating systemic immune activation. Therefore, the dramatic response observed in two melanoma models suggests that MUSIC's use as an adjuvant could improve patient responses to ICIs in cancers where it is the current standard of care.

## 2 Results and Discussion

**Figure 1:**
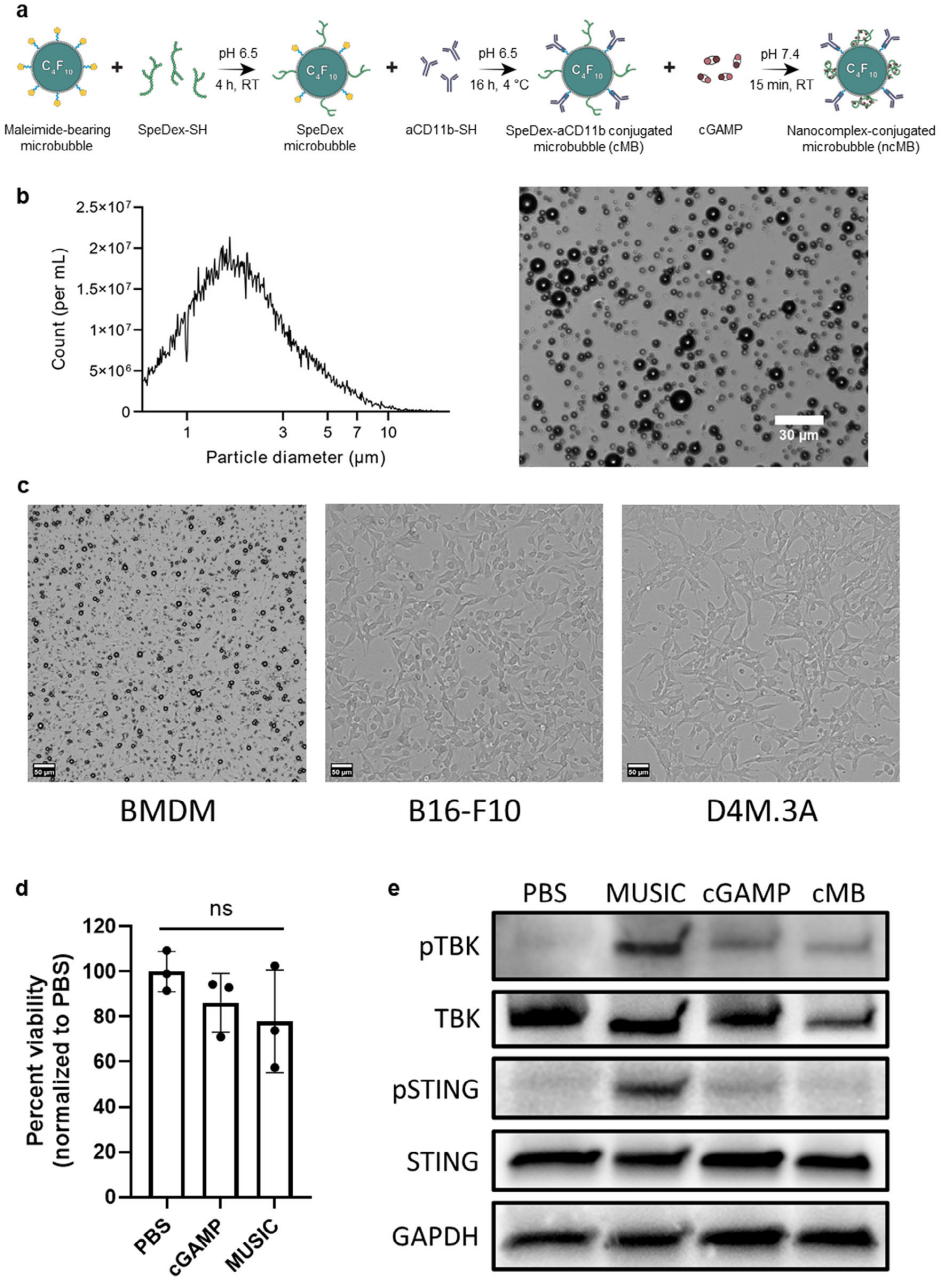
Formulation and characterization of ncMBs. **a**) ncMBs were obtained by conjugating SpeDex‐SH and aCD11b‐SH onto maleimide‐bearing MBs followed by electrostatic binding of cGAMP. **b**) Representative Coulter counter measurements (left) and brightfield microscopy (right) of ncMBs show an average size of 2.3 µm. **c**) Brightfield microscopy of BMDM, B16‐F10, and D4M.3A cells after incubation with CD11b‐targeted ncMBs validates targeting‐specificity. Scale bar is 50 um. **d**) The effect of MUSIC on the viability of BMDMs was determined using the WST‐8 assay 24 h after treatment. Data represent mean ± s.d. and were analyzed by one‐way ANOVA. **e**) BMDMs were treated as indicated, followed by western blotting to assess phosphorylation of proteins in the STING pathway.

We first formulated perfluorobutane (PFB)‐filled microbubbles (MBs) containing maleimide‐bearing end groups (mal‐MBs) via the thin‐film hydration method and tip sonication.^[^ ^18^ ^]^ As unconjugated MBs lack drug‐binding capacity, we synthesized a cationic and thiolated spermine‐modified dextran (SpeDex‐SH) to facilitate cGAMP loading (Figure S1a, Supporting Information).^[^ ^19^ ^]^ The thiol groups enabled conjugation onto the maleimide end groups of mal‐MBs via the thiol‐ene click reaction, forming SpeDex MBs.^[^ ^20^ ^]^ To enable targeting to antigen‐presenting cells (APCs), thiolated anti‐CD11b antibody (aCD11b‐SH, Figure S1b,c, Supporting Information) was then conjugated onto SpeDex MBs to afford SpeDex‐aCD11b MBs (cMBs) with ≈200000 antibodies per bubble (Figure S1d, Supporting Information).^[^ ^21^ ^]^ cMBs were then loaded with cGAMP via electrostatic binding to form ncMBs with an average size of 2.3 µm (**Figure** **1**a,b). The formulation of ncMBs was highly efficient, with nearly all ncMBs conjugated with aCD11b and loaded with cGAMP, as confirmed by flow cytometry and microscopy (Figure S2a,b, Supporting Information). Zeta potential measurements further validated cGAMP loading, showing a drop in surface charge from +4.4 mV for cMBs to ‐5.3 mV for ncMBs (Figure S2c, Supporting Information). Additionally, ncMBs maintained US responsiveness, as observed in US contrast images of intratumorally‐injected ncMBs before and after sonoporation (Figure S2d, Supporting Information). To confirm targeting specificity, ncMBs were incubated with CD11b‐expressing mouse bone marrow‐derived macrophages (BMDMs) and non‐CD11b‐expressing B16F10 and D4M.3A melanoma cells. Brightfield microscopy confirmed the presence of numerous cell‐ncMB complexes after incubation with BMDMs, while no bound ncMBs were observed after incubation with B16F10 or D4M.3A cells (Figure 1c). To assess the toxicity of ncMBs on BMDMs after sonoporation (MUSIC), metabolic activity was measured using the WST‐8 assay 24 hours after treatment, which showed a slight, but not statistically significant, decrease in metabolic activity (Figure 1d). Finally, activation of the STING pathway was validated by western blot (Figure 1e). Results show that MUSIC produced greater phosphorylation of STING and TANK‐binding kinase 1 (TBK‐1) in BMDMs compared to cGAMP alone, suggesting improved cytosolic delivery of cGAMP.^[^ ^22^ ^]^ Collectively, these results demonstrate that ncMBs exhibit high cGAMP loading, specific targeting to APCs, and improved delivery of cGAMP into macrophages upon sonoporation with no sign of acute toxicity.

**Figure 2:**
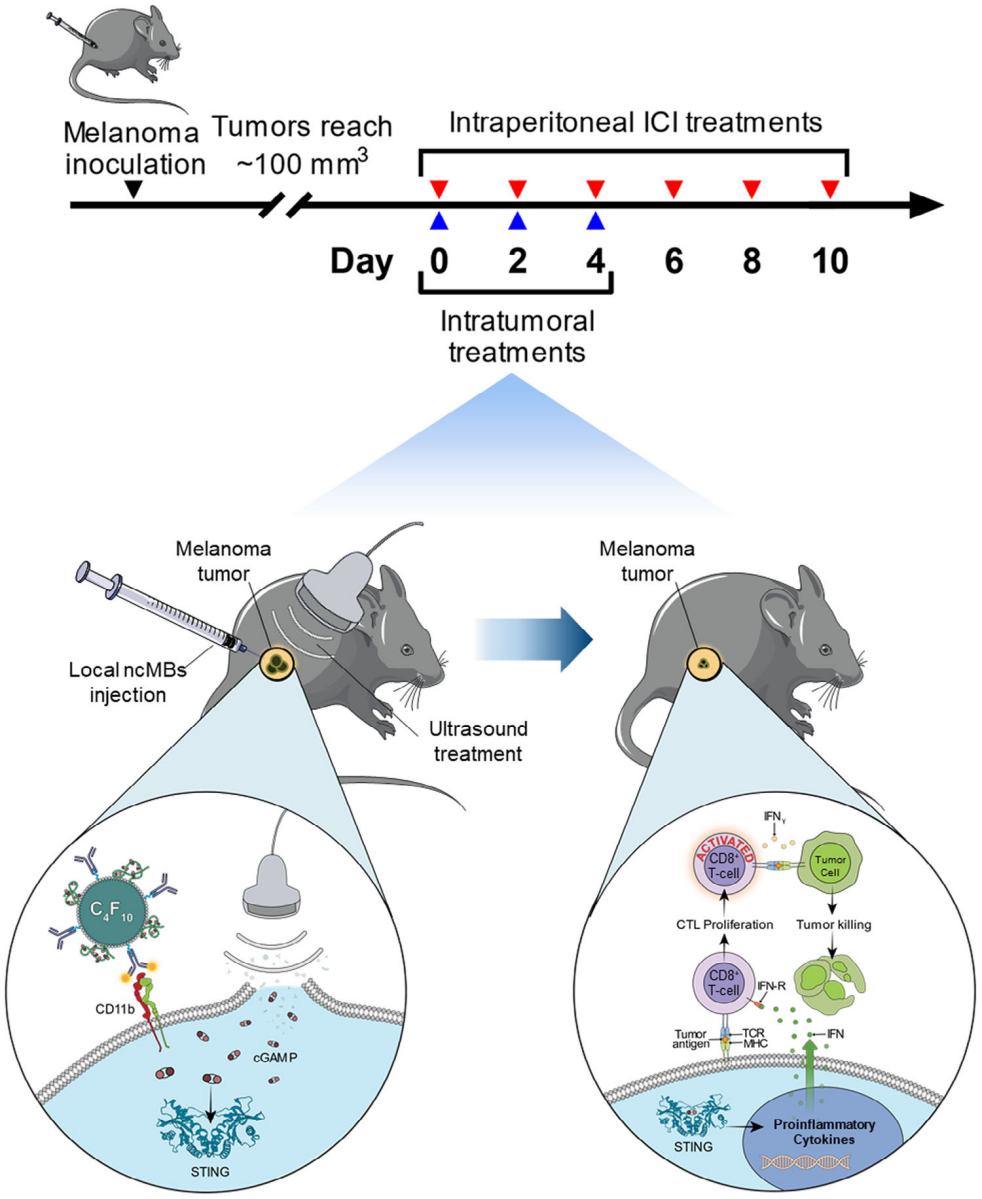
Experimental design for MUSIC treatment of melanoma. Mice were inoculated with melanoma tumors that grew to ≈100 mm^3^. In conjunction with ICI therapy, mice then underwent MUSIC treatment to deliver cGAMP into the cytosol of tumor‐associated APCs. The resulting STING activation led to the production of downstream interferons that assisted in T‐cell activation and tumor killing.

**Figure 3:**
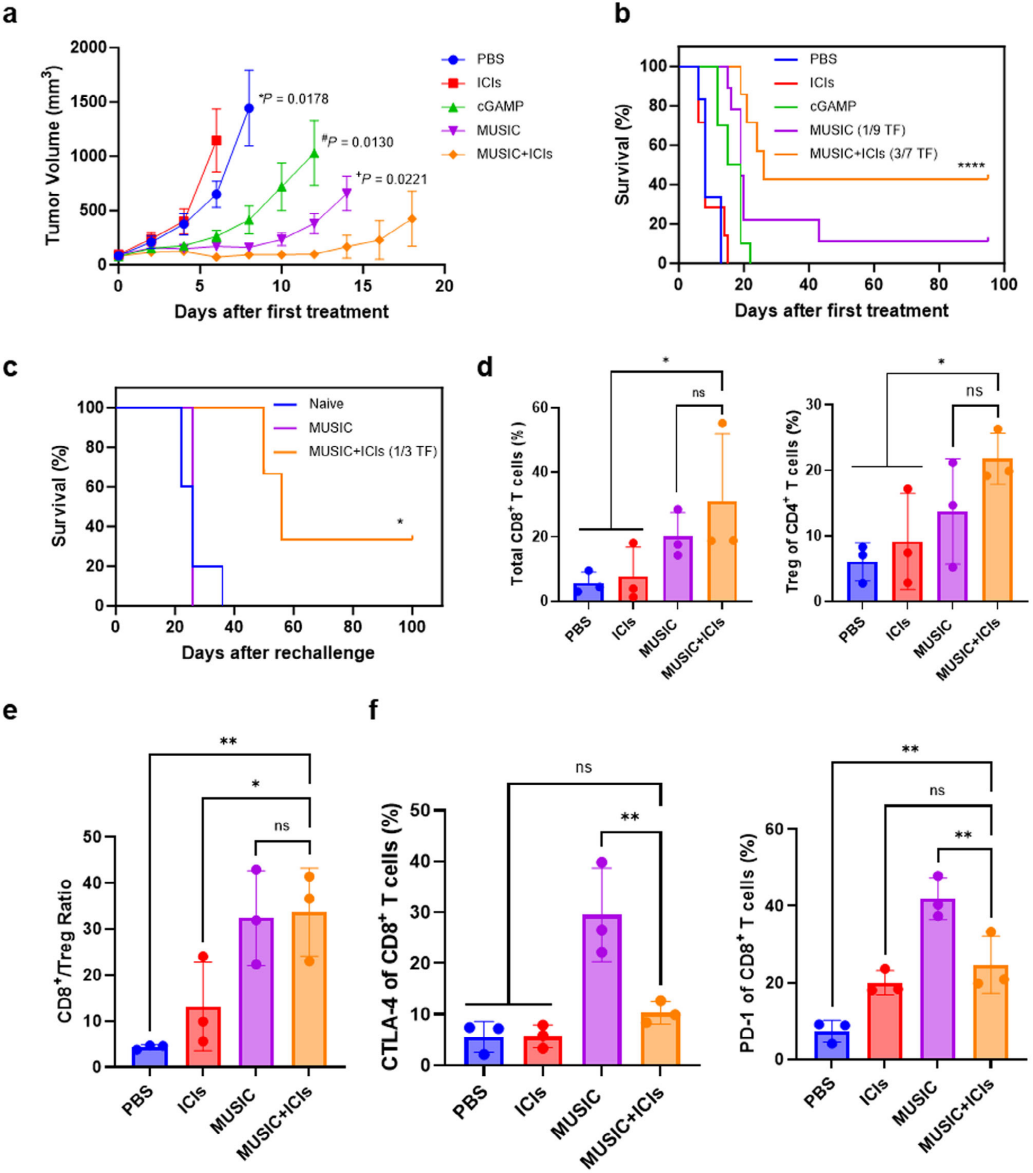
MUSIC treatment of B16‐F10 melanoma. **a**) Tumor volumes were monitored and analyzed over the indicated periods. **b**) Mouse survival for the different groups was monitored for 95 days. *n* = 6 (PBS), 7 (ICIs, MUSIC+ICIs), 9 (MUSIC), and 10 (cGAMP). **c**) The resulting tumor‐free mice were then rechallenged with B16‐F10 cells. Tumor volumes were then monitored for the next 100 days. *n* = 5 (Naïve), 1 (MUSIC), and 3 (MUSIC+ICIs). **d**) Flow cytometry quantification of CD8^+^ T cells and Tregs in tumors 24 hours after the third treatment. **e**) Ratio of the number of CD8^+^ T cells to Tregs. **f**) Number of CD8^+^ T cells from (**d**) that express CTLA4 (left) and PD1 (right). n = 3 replicates for all groups. The data represent mean ± s.e.m. (**a**) or mean ± s.d. (**d,e,f**). Data were analyzed by multiple unpaired t tests with Welch correction (**a**), two‐sided log‐rank (Mantel–Cox) test (**b,c**), or one‐way ANOVA with Fisher's LSD (**d,e,f**). *P, ^#^P, and *^+^P* denote the statistical comparison of MUSIC+ICIs versus PBS, MUSIC+ICIs versus cGAMP, and MUSIC+ICIs versus MUSIC, respectively. *p* values > 0.05 were considered not significant (ns), *p* values < 0.05 were considered significant. *p value < 0.05, **p value < 0.01, ***p value < 0.001, ****p value < 0.0001.

Because of the results obtained in our previous study with murine breast cancer models, we hypothesized that MUSIC would synergize with ICIs, recruit and prime T cells, enhance tumor growth inhibition, and improve survival in melanoma as well (**Figure** **2**). To assess our hypothesis, we investigated the antitumor immune responses generated by MUSIC against the *Braf^wt^* B16‐F10 and *Braf^V600E^* D4M.3A murine melanoma tumor models.^[^ ^23^, ^24^ ^]^ Monitoring the B16‐F10 tumor‐bearing mice for 95 days after treatment revealed that the combination of MUSIC and ICIs resulted in the greatest tumor growth inhibition and survival benefit compared to all other groups (**Figure** **3**a,b). Additionally, complete tumor eradication was observed in three out of the seven combination‐treated mice and one out of the nine MUSIC‐treated mice (Figure S3a, Supporting Information). To evaluate the establishment of immune memory, these tumor‐free (TF) mice were rechallenged with the same B16‐F10 cell line by inoculating them on the flank contralateral to the original implantation site (Figure S4, Supporting Information). While two out of the three combination‐treated mice grew tumors after rechallenging, their median survival time was over double that of the naïve group, suggesting a much better immune response (Figure 3c). Notably, a single combination‐treated mouse remained TF for at least 100 days after rechallenging, indicating that the combination treatment produced systemic antitumor memory responses and long‐term immunity. The rechallenged MUSIC‐treated mouse also grew a tumor, but with no significant difference in its survival time compared to that of the naïve group. The difference in survival times between MUSIC‐treatment and combination‐treatment suggests that the combination of MUSIC and ICIs resulted in a better systemic immune response than MUSIC alone. The combination of MUSIC and ICIs was also well‐tolerated, as liver and kidney panels did not show any statistically significant differences in aspartate transferase (AST), alanine transferase (ALT), alkaline phosphatase (ALP), and albumin levels; however, a slight increase in blood urea nitrogen (BUN) was observed following multiple treatments (Figure S5, Supporting Information).

To quantify the effects of MUSIC on the immune landscape, treatments were repeated on a separate cohort of mice, and tumors were collected 24 hours after the third treatment for staining and flow cytometry (Figure S6a, Supporting Information). Staining for CD8^+^ T cells and regulatory T cells (Treg) showed that MUSIC with ICIs increased the presence of both cell types in the tumor compared to control groups (Figure 3d). While the difference between MUSIC alone and MUSIC with ICIs was not significant, the percentage of both cell populations trended higher with the combination treatment. The observed increase in Tregs was also consistent with previous reports showing that checkpoint blockade with aCTLA4 causes hyper‐proliferation of Tregs in tumor tissue.^[^ ^25^ ^]^ It is also well known that a high Treg count in tumors is associated with poor prognosis;^[^ ^26^, ^27^, ^28^ ^]^ however, it is apparent that the ratio of CD8^+^ T cells to Tregs is more relevant, with higher ratios correlating to better clinical outcomes.^[^ ^29^, ^30^, ^31^ ^]^ Surprisingly, MUSIC treatment with or without ICIs resulted in a similar CD8/Treg ratio, with both groups having a significantly increased ratio compared to control groups. These results prompted us to examine the expression of CTLA4 and PD1 on the infiltrating CD8^+^ T cells to explain the differences between the antitumor responses of these two groups. Since activation of naïve T cells results in upregulation of the inhibitory CTLA4 and PD1 receptors as a means of maintaining T cell homeostasis, it is possible that these receptors were dampening the immune response from MUSIC.^[^ ^32^ ^]^ We observed that MUSIC resulted in an increased number of CD8^+^ T cells expressing CTLA4 and PD1, which was curtailed with the addition of ICIs (Figure 3f). We reasoned then that MUSIC was indirectly upregulating the immune checkpoints through increased T cell activation. The addition of ICIs then inhibited these suppressive signals and resulted in a synergistically greater antitumor response.

**Figure 4:**
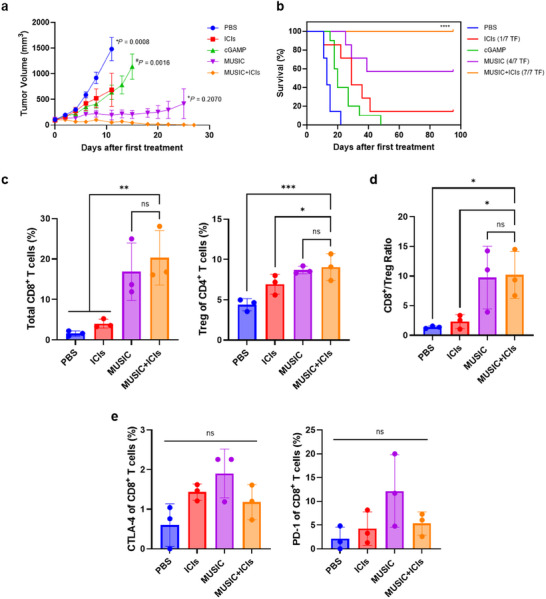
MUSIC treatment of D4M.3A melanoma. **a**) Tumor volumes were monitored and analyzed over the indicated periods. **b**) Mouse survival for the different groups was monitored for 95 days. *n* = 7 (PBS, ICIs, MUSIC, MUSIC+ICIs) and 10 (cGAMP). **c**) Flow cytometry quantification of CD8^+^ T cells and Tregs in tumors 24 hours after the third treatment. **d**) Ratio of the number of CD8^+^ T cells to Tregs. **e**) Number of CD8^+^ T cells from (**c**) that express CTLA4 (left) and PD1 (right). *n =* 3 biological replicates for all groups. The data represent mean ± s.e.m. (**a**) or mean ± s.d. (**c,d,e**). Data were analyzed by multiple unpaired t tests with Welch correction (**a**), two‐sided log‐rank (Mantel–Cox) test (**b**), or one‐way ANOVA with Fisher's LSD (**c,d,e**). *P, ^#^ *P*, and ^+^ *P* denote the statistical comparison of MUSIC+ICIs versus PBS, MUSIC+ICIs versus cGAMP, and MUSIC+ICIs versus MUSIC, respectively. *p* values > 0.05 were considered not significant (ns), *p* values < 0.05 were considered significant. *p value < 0.05, **p value < 0.01, ***p value < 0.001, ****p value < 0.0001.

While results obtained with the B16‐F10 model were encouraging, we observed a much greater tumor inhibitory effect in the D4M.3A model, achieving complete tumor eradication in four out of the seven mice treated with MUSIC. Inclusion of ICIs with MUSIC resulted in a 100% survival rate, showing great synergy between the two treatments (**Figure** **4**a,b; Figure S3b, Supporting Information). Similar to the B16‐F10 model, MUSIC treatment with or without ICIs did not result in a significant difference in the number of CD8^+^ T cells or Tregs (Figure 4c). Surprisingly however, the CD8/Treg ratio was two folds lower in the D4M.3A tumors than in the B16‐F10 tumors, with no significant difference observed between MUSIC with or without ICIs (Figure 4d). The difference in the number of CD8^+^ T cells expressing CTLA4 and PD1 was also statistically non‐significant between all groups, even though the levels did trend higher with MUSIC treatment (Figure 4e). This higher trend could potentially be why MUSIC alone did not show complete tumor eradication in all mice, even though the response was relatively efficacious.

Given its *Braf* mutation, D4M.3A's increased response to MUSIC suggests that certain mutations or characteristics could make tumors more susceptible to this treatment, even though MUSIC targets the host's immune system and not the cancer itself. Similar to what we observed, it has been reported that D4M.3A tumors respond better to STING activation and checkpoint inhibition than B16‐F10 tumors, potentially through STING‐mediated downregulation of nuclear factor (erythroid‐derived 2)‐like 2 (NRF2).^[^ ^33^ ^]^ NRF2 is a transcription factor responsible for regulating anti‐oxidative response against reactive oxidative species and protects against oxidative damage, thus indirectly acting as a mediator of oncogenesis.^[^ ^34^ ^]^ High constitutive BRAF expression, such as in D4M.3A, results in increased NRF2 expression that amplifies cell proliferation.^[^ ^35^ ^]^ However, type‐I interferons prevent NRF2 from translocating into the nucleus to induce expression of antioxidant proteins.^[^ ^36^ ^]^ Therefore, the type‐I interferons produced from MUSIC are possibly disrupting NRF2 activity and thus reducing the viability of the D4M.3A tumor. Furthermore, B16‐F10 tumors have been shown to proliferate at statistically similar rates in *Nrf^+/+^* mice as in *Nrf^−/−^* mice, suggesting that NRF2 downregulation is not as important in this model as it is in D4M.3A and potentially explaining the better efficacy observed from MUSIC in D4M.3A.^[^ ^37^ ^]^ The lower CD8^+^ T cell activity of D4M.3A tumors when compared to B16‐F10 tumors after MUSIC treatment coupled with the significant increase in interferon‐producing CD11b^+^ cells in the tumor also suggests that these interferon‐producing cells play an important role in the antitumor efficacy in D4M.3A tumors (Figure S6b,c, Supporting Information).

## 3 Conclusion

Our study presents MUSIC as a promising strategy for enhanced immunotherapy against melanoma, further demonstrating its potential as an immune adjuvant to the standard of care immunotherapeutics. Combined administration of MUSIC and ICIs showed superior efficacy in inhibiting tumor growth and enhancing curative rates in two models of murine melanoma, underscoring the synergy between MUSIC and T cell‐based checkpoint inhibition. MUSIC especially showed efficacious response against the D4M.3A model, which harbors a clinically relevant BRAF mutation that appears in 50% of melanoma patients.^[^ ^38^ ^]^ This suggests that, although MUSIC's mechanism of action is STING‐dependent, certain tumor‐specific mutations can dictate the response to MUSIC even within the same cancer indication. This study holds significant translational potential, since the major subcomponents of MUSIC are FDA‐approved, and because these findings provide insight for determining the optimal responders to MUSIC in the clinic.

## 4 Experimental Section

### Materials

1,2‐distearoyl‐sn‐glycero‐3‐phosphocholine (DSPC) and 1,2‐distearoyl‐sn‐glycero‐3‐phosphoethanolamine‐N‐[methoxy(polyethylene glycol)‐2000] (DSPE‐PEG2k) were purchased from NOF America. 1,2‐distearoyl‐sn‐glycero‐3‐phosphoethanolamine‐N‐[maleimide(polyethylene glycol)‐5000] (DSPE‐PEG5k‐mal) was purchased from NANOCS. Perfluorobutane (PFB) was purchased from Fluoromed. Dextran‐40, spermine, sodium borohydride, 2‐iminothiolane, dialysis tubing, Amicon Ultra‐0.5 centrifugal filter units, DMEM, penicillin‐streptomycin, RIPA buffer, phosphatase inhibitor, protease inhibitor, PVDF membranes, sinapic acid, and trifluoroacetic acid were purchased from Sigma‐Aldrich. Potassium periodate, borate buffer, DMEM/F12, non‐essential amino acids solution (NEAA), FBS, mouse macrophage colony‐stimulating factor (M‐CSF), SuperSignal West Femto Maximum Sensitivity Substrate, Bolt LDS sample buffer, Bolt Sample Reducing Agent, PageRuler Plus Prestained Protein Ladder, Bolt Bis‐Tris Plus Mini Protein Gels, Bolt MES SDS running buffer, and SilverQuest Silver Staining Kit were purchased from ThermoFisher. cGAMP was purchased from Chemietek. DY547‐c‐diGMP was purchased from Enzo. Anti‐mouse/human CD11b antibody (clone M1/70) and Alexa Fluor 647 anti‐mouse/human CD11b antibody (clone M1/70) were purchased from BioLegend. WST‐8 was purchased from Abcam. Mini‐PROTEAN TGX Stain‐Free Gels, Tris/Glycine/SDS Buffer, TBST, and milk were purchased from BioRad. Serum Separator Microtainer tubes were purchased from BD. B16‐F10 cells (RRID:CVCL_0159) were purchased from ATCC. D4M.3A cells (RRID:CVCL_0P27) were a gift from Dr. David Fisher (Massachusetts General Hospital).^[^ ^39^ ^]^ C57Bl/6 mice were purchased from Jackson Laboratories.

### Synthesis of SpeDex

SpeDex polymer was synthesized as previously reported.^[^ ^19^ ^]^ Briefly potassium periodate (1420 mg, 6.17 mmol) was added to a solution of dextran‐40 (1000 mg, 6.17 mmol of glucose monomers) in milliQ water (20 mL). The reaction was vigorously stirred in the dark for 7 h at room temperature and then dialyzed for 24 h against water using a regenerated cellulose dialysis tubing (MWCO 3.5–5 kDa). The oxidized dextran was then added to a solution of spermine (473 mg, 3.07 mmol) in borate buffer (20 mL, 0.1 M, pH 11) over 5 h via syringe pump. The resulting solution was stirred for 24 h at room temperature followed by addition of NaBH4 (371 mg, 9.82 mmol) under ice bath and further stirring for 48 h at room temperature. An additional portion of NaBH4 (371 mg, 9.82 mmol) was then added and stirring continued for 24 h under the same conditions. Crude product was dialyzed against water (MWCO  =  3.5–5 kDa) for 48 h followed by lyophilization for 48 h to yield SpeDex. For thiolation, SpeDex (242 mg) was dissolved with 1× PBS containing 5 mmol L^−1^ ethylenediaminetetraacetic acid (EDTA, 24.2 mL) to obtain a 10 mg mL^−1^ solution. To this, a 1 mg mL^−1^ aqueous solution of 2‐iminothiolane HCl (23.3 mg, 169.6 µmol) was added dropwise with vigorous stirring. The resulting mixture was stirred for 1 h, dialyzed for 48 h (MWCO  =  3.5 kDa), and lyophilized for 48 h to yield SpeDex‐SH.

### MB Formulation and SpeDex conjugation

MBs were formulated as previously reported.^[^ ^18^ ^]^ Briefly, lipid films were prepared by dissolving a mixture of DPSC (14.37 mg, 18.18 µmol), DSPE‐PEG2k (2.83 mg, 1.01 µmol) and DSPE‐PEG5k‐mal (6 mg, 1.01 µmol) lipids in chloroform (750 µL) at a 90:5:5 molar ratio and slowly evaporating with a rotary evaporator (Büchi Rotavapor R‐100) until mostly dry. The resulting films were then further dried overnight under vacuum and stored at −20 °C for later use. The lipid films were solvated in a mixture of PBS 1×/propylene glycol/glycerol (80:10:10 v/v/v, 10 mL total) and bath sonicated at 70 °C until clear or for 15 min. PFB vapor was then introduced into the solution, and the resulting mixture was tip sonicated at 70% amplitude for five seconds before being cooled down in an ice bath. The resulting MB formulation was washed three times with PBS 1× pH 6.5 plus 1 mmol L^−1^ EDTA using centrifugation (300*g* , 3 min) to yield PEGylated MBs with terminal maleimide functions (mal‐MBs). SpeDex‐SH was then dissolved in the same PBS solution at 10 mg mL^−1^ and added to mal‐MBs at a 1:20 maleimide:SpeDex molar ratio. The solution was rotated end‐over‐end for 4 h then washed three times with PBS 1× pH 6.5 plus 1 mmol L^−1^ EDTA (300*g* , 3 min) to yield SpeDex MBs.

### Antibody Thiolation and Preparation of ncMBs

Anti‐CD11b (aCD11b, Biolegend 101 202) was first thiolated to allow for conjugation onto maleimide‐bearing SpeDex MBs. Briefly, a 2 mg mL^−1^ solution of 2‐iminothiolane in PBS pH 8.0 with 5 mmol L^−1^ EDTA was added at a 600:1 molar ratio to a solution of aCD11b in PBS with 5 mmol L^−1^ EDTA. This solution was rotated for 2 h before having its buffer exchanged with PBS pH 6.5 with 1 mmol L^−1^ EDTA by using an Amicon Ultra centrifugal filter (10 kDa MWCO). The number of thiols per antibody was quantified using matrix‐assisted laser desorption ionization–time of flight (MALDI‐TOF) spectroscopy (Voyager DE‐Pro) with a sinapic acid matrix dissolved in a 50:50 water:acetonitrile buffer containing 0.1% trifluoroacetic acid. SpeDex MBs were then added to the solution of aCD11b‐SH at a ratio of 0.2 equivalents of antibody per maleimide and rotated end‐over‐end for 16 h at 4 °C to allow for conjugation. Afterwards, the solution was washed three times with PBS at 300*g*  for 3 min to afford SpeDex‐aCD11b‐conjugated MBs (cMBs). cMBs were then incubated with cGAMP for 15 minutes to obtain nanocomplex‐conjugated MBs (ncMBs).

### MB Characterization

MB size, concentration, and zeta potential were obtained at initial formulation and after every conjugation step. Size and concentration were obtained using a Coulter Counter (Beckman Coulter Multisizer 4) and brightfield microscopy (Zeiss Axio A1 upright microscope). Zeta potential was measured with the Malvern Zetasizer Nano ZS using 1 mM NaCl with 10% v/v glycerol as running buffer. The number of aCD11b antibodies per cMB was quantified using SDS‐PAGE. cMBs were first bath sonicated, mixed with Bolt LDS buffer and reducing agent, heated at 70 °C for 30 min, loaded in Bolt Bis‐Tris Plus Mini gels, and subjected to electrophoresis at 80 V using Bolt MES SDS running buffer. The gel was then stained using the Invitrogen SilverQuest Silver Staining Kit and imaged (BioDoc‐It Gel Imaging System). Band intensities were quantified using ImageJ and compared to a standard curve made from aCD11b. To quantify the percentage of cMBs conjugated with aCD11b, AlexaFluor 647 anti‐CD1b (AF647‐aCD11b, Biolegend 101 220) was used as a fluorescent analog. Thiolation and conjugation procedures were repeated as described above, followed by analysis using flow cytometry (BD Accuri C6). To confirm cGAMP binding onto cMBs, DY547‐c‐diGMP was used as a CDN fluorescent analog. After binding DY547‐c‐diGMP, ncMBs were imaged using fluorescent microscopy (Zeiss Axio A1 upright microscope with Olympus TRITC filter cube) and analyzed using flow cytometry.

### Cell Culture

Cell cultures were incubated at 37 °C in humidified conditions equilibrated with 5% CO_2_. B16‐F10 cells were maintained in DMEM containing high glucose supplemented with 10% FBS. D4M.3A cells were maintained in DMEM/F12 (1:1) containing L‐glutamine and sodium bicarbonate supplemented with 10% FBS and 1% NEAA. Mouse BMDMs were isolated from the bone marrow of the femurs and tibias of C57Bl/6 mice and cultured with M‐CSF (20 ng ml^−1^) in DMEM supplemented with 10% FBS and 1% penicillin‐streptomycin according to a previously reported procedure.^[^ ^40^ ^]^

### In vitro *Studies*

ncMBs loaded with 10 nmol of cGAMP were added to cultures of BMDMs, B16F10, and D4M.3A cells in 12‐well plates at a ratio of 10 ncMBs per cell and incubated upside down without media for 10 min to allow for targeting. Afterwards, wells were washed and filled with 2.5 mL of PFB‐saturated media and imaged with brightfield microscopy (BioTek Cytation 5 Cell Imager) to confirm targeting specificity. BMDM cells that received ncMBs were then sonoporated (MUSIC) from the top using a 1 MHz plane wave transducer operating at 2 W cm^−2^ for 60 seconds at a 50% duty cycle (GTS Sonitron) followed by collection after 6 h for western blotting. PBS, cGAMP, and cMBs + US were used as controls. A separate set of BMDMs was treated with MUSIC, cGAMP, and PBS followed by incubation with WST‐8 after 24 h to measure cell viability (Biotek Synergy H1 Plate Reader).

### Western Blot

Cells were lysed with radioimmunoprecipitation assay (RIPA) lysis buffer containing a protease and phosphatase inhibitor cocktail according to the manufacturer's instructions. Protein samples were collected from the supernatants and concentrations quantified by the bicinchoninic acid assay (BCA) assay. Samples were boiled for 5 min, loaded in Mini‐PROTEAN TGX Stain‐Free gels at equal amounts, and subjected to electrophoresis (80 V) using Tris/Glycine/SDS running buffer. Proteins were then transferred to polyvinylidene difluoride (PVDF) membranes and blocked using 5% non‐fat milk in TBST for 1 h at room temperature. Membranes were then incubated overnight at 4 °C with primary antibodies, washed with TBST, and then incubated with HRP‐conjugated secondary antibody for 1 h at room temperature. Membranes were washed again and incubated with SuperSignal West Femto Maximum Sensitivity Substrate for 5 min before being imaged (BioRad ChemiDoc Imaging System). Primary antbodies included TBK (clone D1B4, Cell Signaling Technology 3504, 1:1000), pTBK (clone D52C2, Cell Signaling Technology 5483, 1:1000), STING (clone D1V5L, Cell Signaling Technology 50 494, 1:1000), and pSTING (clone D8F4 W, Cell Signaling Technology 72 971, 1:1000). HRP‐linked anti‐rabbit IgG (Cell Signaling Technology 7074, 1:5000) was used as the secondary antibody. GAPDH (clone 14C10, Cell Signaling Technology 2118, 1:1000) was used as a loading control.

### Animal Studies

All mice were maintained at the institution's animal facility in a specific pathogen‐free environment with an ambient temperature of 22 °C and a relative humidity of 50%. All mice were maintained on a standard diet and water in a 12 h/12 h light/dark cycle. All animal experiments were approved by the Institutional Animal Care and Use Committee of the University of Texas Southwestern Medical Center under the animal protocol #2020‐102971. Age‐matched male C57Bl/6 mice (6–8 weeks, Jackson Laboratory) were used for all animal experiments. For tumor engraftment, mice were anesthetized with 2% isoflurane in O_2_ before having their right flank shaved and depilated. 100 µL of cell solution (100000 B16‐F10 cells per mouse or 300000 D4M.3A cells per mouse) were then injected subcutaneously into their right flank. Tumors were measured with calipers every other day, and tumor volumes were calculated according to an ellipsoid formula (1/2 × length × width^2^). When tumors reached approximately 100 mm^3^, mice were randomized into groups of 6–10 and injected intratumorally with PBS, cGAMP (100 µg), or ncMBs (2.7×10^7^ cMBs loaded with 100 µg cGAMP) as a 20 µL solution every other day for three days. ncMB‐injected mice were then sonoporated (MUSIC) on two sides of the tumor using a 1 MHz plane wave transducer operating at 4 W cm^−2^ for 60 seconds at a 50% duty cycle (GTS Sonitron). Groups receiving ICIs were injected intraperitoneally with a 200 µL solution of aCTLA4 (200 µg) and aPD1 (200 µg) mixed together every other day for six days. A separate cohort was used to evaluate systemic toxicity of MUSIC as a mono therapy or when combined with ICIs. For these experiments, mice were treated with PBS, MUSIC or MUSIC + ICIs as described above. 24 h after the final treatment, blood was collected from the submandibular vein into microtainer serum separator tubes for chemistry analysis (Roche Integra 400 Plus).

### Immune Cell Quantification

Tumor tissues were carefully resected 24 hours after the third treatment and dissociated using a mouse tumor dissociation kit (Miltenyi). Flow cytometry analysis was conducted with a Gallios flow cytometer (Beckman Coulter). Fluorescence‐labelled antibodies included CD25 (clone PC61.5, eBioscience 79‐0251‐82, 1:500 dilution), CD8 (clone 53–6.7, BioLegend 100 722, 1:250), CD4 (clone GK1.5, BioLegend 100 408, 1:250), FoxP3 (clone FJK‐16s, eBioscience 11‐57723‐82, 1:500), CD3 (clone 17A2, BioLegend 100 222, 1:250), CTLA4 (clone UC10‐4B9, BioLegend 106 315, 1:250), CD45 (clone HI30, BioLegend 304 024, 1:500), PD‐1 (clone 29F.1A12, BioLegend 135 228, 1:250), and CD11b (clone M1/70, BioLegend 101 224, 1:500). A LIVE/DEAD fixable red dead cell stain kit (Sytox Red, Invitrogen S34859, 1:1000) was used to stain dead cells.

### Statistical Analysis

Statistical analysis was performed using Graphpad Prism Version 10.0. Values reported in figures were expressed as mean ± standard error of the mean (s.e.m) or mean ± standard deviation (s.d.) from at least three biological replicates, unless otherwise indicated. Statistical analysis included multiple unpaired t tests with Welch correction and one‐way analysis of variance (ANOVA) followed by Fisher's Least Significant Difference (LSD) test. Survival analysis was conducted using the Kaplan–Meier estimator and compared by the two‐sided log‐rank (Mantel–Cox) test. The sample size for each statistical analysis was presented in figure legend. *p* values > 0.05 were considered not significant (ns), *p* values < 0.05 were considered significant. *p value < 0.05, **p value < 0.01, ***p value < 0.001, ****p value < 0.0001.

## Conflict of Interest

J.L., W.J., and S.K. were cofounders of MusiQ Bio. J.L and W.J. are members of the Scientific Advisory Board of MusiQ Bio.

## Supporting information



Supporting Information

## Data Availability

The data that support the findings of this study are available from the corresponding author upon reasonable request.
