# Supplementary material for: Targeted STING Activation Using Modified Ultrasound‐Responsive Microbubbles Enhances Immune Checkpoint Blockade Against Melanoma
Source: Adv Sci (Weinh). 2025 Mar 26;12(22):2416596. doi: 10.1002/advs.202416596 (PMC12165055; doi:10.1002/advs.202416596)
Supplement: Supplementary file 1 — Supporting Information [file ADVS-12-2416596-s001.docx]

Supporting Information

Targeted STING Activation using Modified Ultrasound-Responsive Microbubbles Enhances Immune Checkpoint Blockade against Melanoma

Sina Khorsandi, Kristin Huntoon, Yifan Wang, Adam Woodward, Abin Antony, Connor Endsley, Nazia Hafeez, Jared L. Edwards, Nicole McCuen, Prasanna G. Alluri, Betty Y.S. Kim, Wen Jiang*, Jacques Lux*

S. Khorsandi, A. Woodward, C. Endsley, N. Hafeez, J. Lux

Department of Radiology

University of Texas Southwestern Medical Center

Dallas, TX 75390, USA
E-mail: [Jacques.Lux@UTSouthwestern.edu](mailto:Jacques.Lux@UTSouthwestern.edu)

J. Lux

Department of Biomedical Engineering

University of Texas Southwestern Medical Center

Dallas, TX 75390, USA

Y. Wang, A. Antony, J. L. Edwards, W. Jiang

Department of Radiation Oncology

MD Anderson Cancer Center

Houston, TX 77030, USA

E-mail: WJiang4@MDAnderson.org

K. Huntoon, B.Y.S. Kim

Department of Neurosurgery

MD Anderson Cancer Center

Houston, TX 77030, USA

N. McCuen, P. G. Alluri

Department of Radiation Oncology

University of Texas Southwestern Medical Center

Dallas, TX 75390, USA


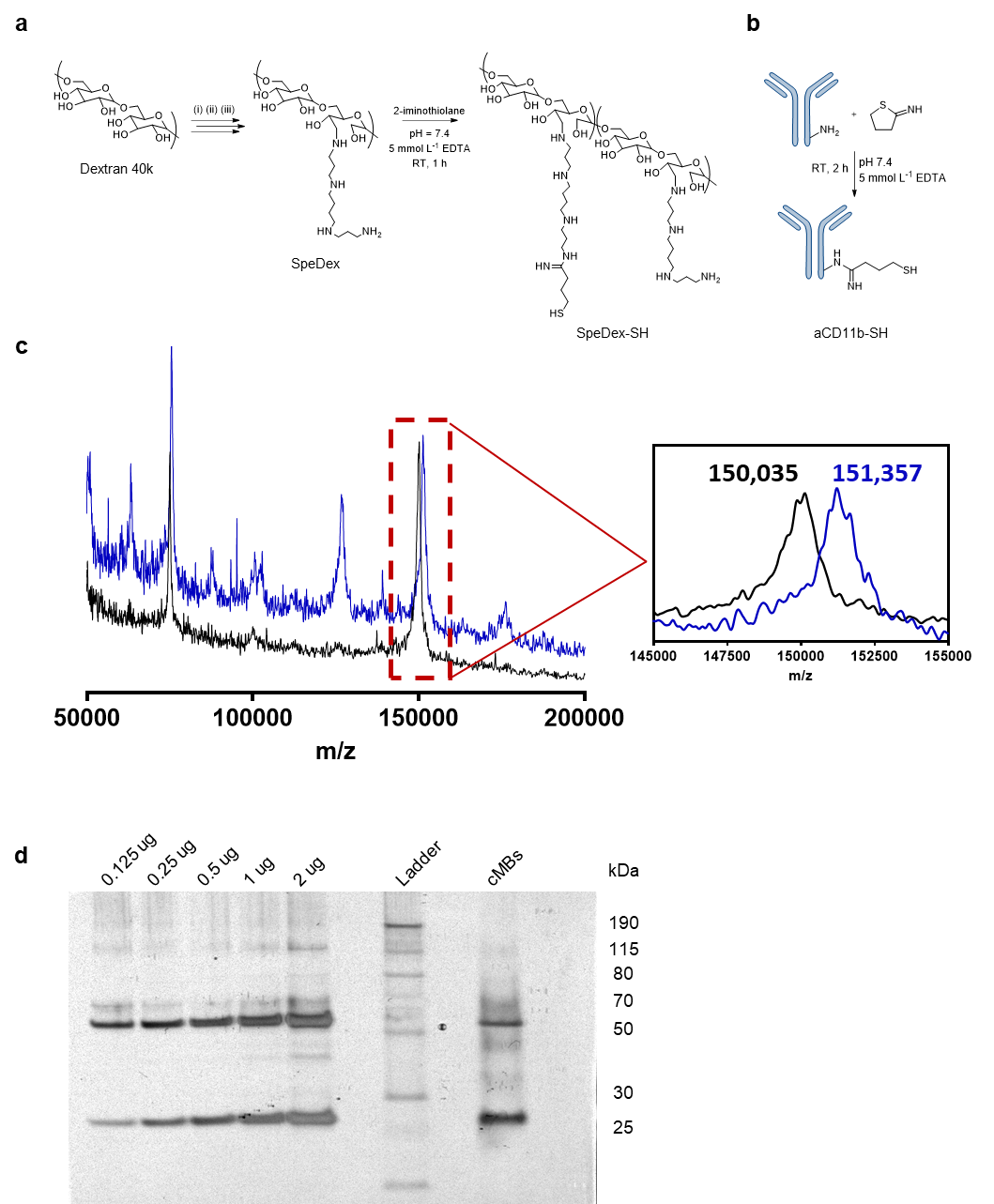


**Figure S1.** Synthesis schemes and characterization of aCD11b. **a**) SpeDex-SH polymer was synthesized via reductive amination of Dextran followed by thiolation with 2-iminothiolane. **b**) aCD11b antibody was thiolated with 2-iminothiolane to yield aCD11b-SH. **c)** MALDI-TOF was used to characterize aCD11b before and after thiolation, represented by the black and blue traces, respectively. The peak shift at 150 kDa corresponds to the addition of 9-10 thiol groups per antibody on average. **d)** The number of aCD11b on cMBs was calculated by running known quantities of aCD11b on an SDS-PAGE gel and measuring the intensities of their bands. Using this standard curve yielded ~209,024 aCD11b antibodies per cMB.


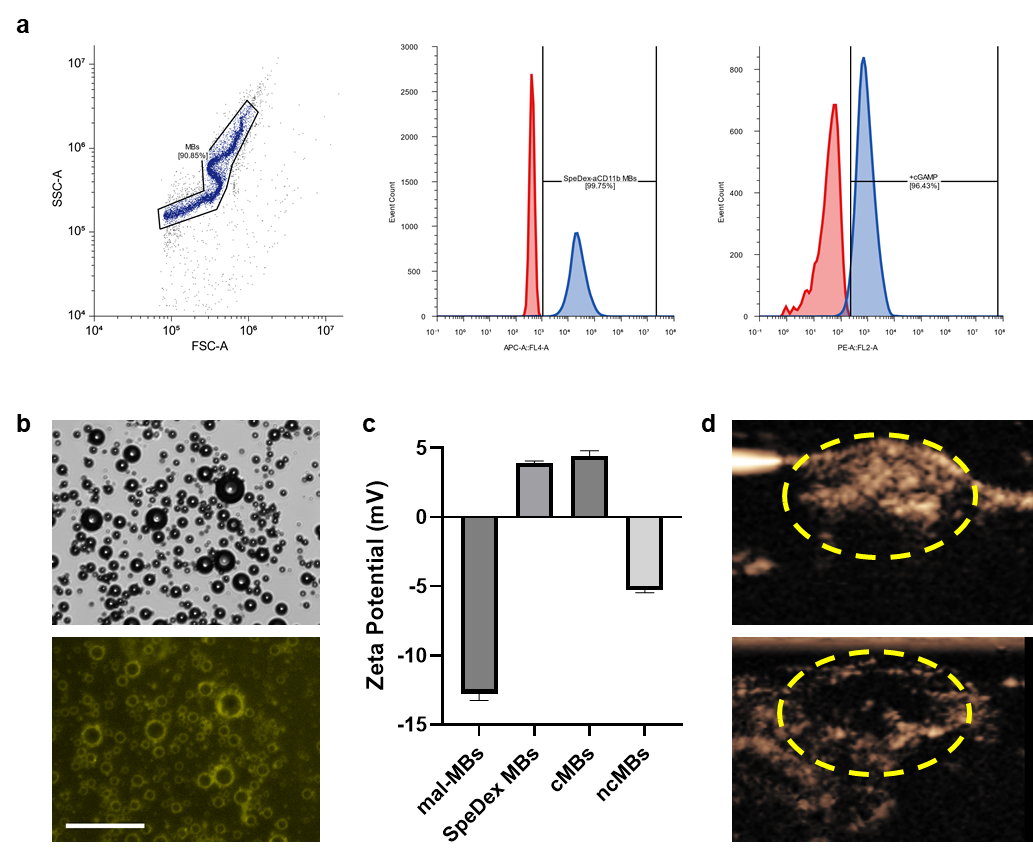


**Figure S2.** Additional characterization of ncMBs. **a**) Flow cytometry characterization of ncMBs after conjugation with APC-aCD11b (center) and loading with fluorescent cGAMP analog (DY547-c-diGMP, right). **b**) Brightfield (top) and fluorescence microscopy (bottom) images of ncMBs loaded with fluorescent cGAMP analog (DY547-c-diGMP). Scale bar is 50 um. **c**) Zeta potential measurements of mal-MBs, SpeDex MBs, cMBs, and ncMBs. The data represent mean ± s.d. with *n* = 3 replicates for all groups. **d**) Contrast-mode ultrasound images of D4M.3A melanoma tumors after injection of ncMBs (top) and after sonoporation (bottom).


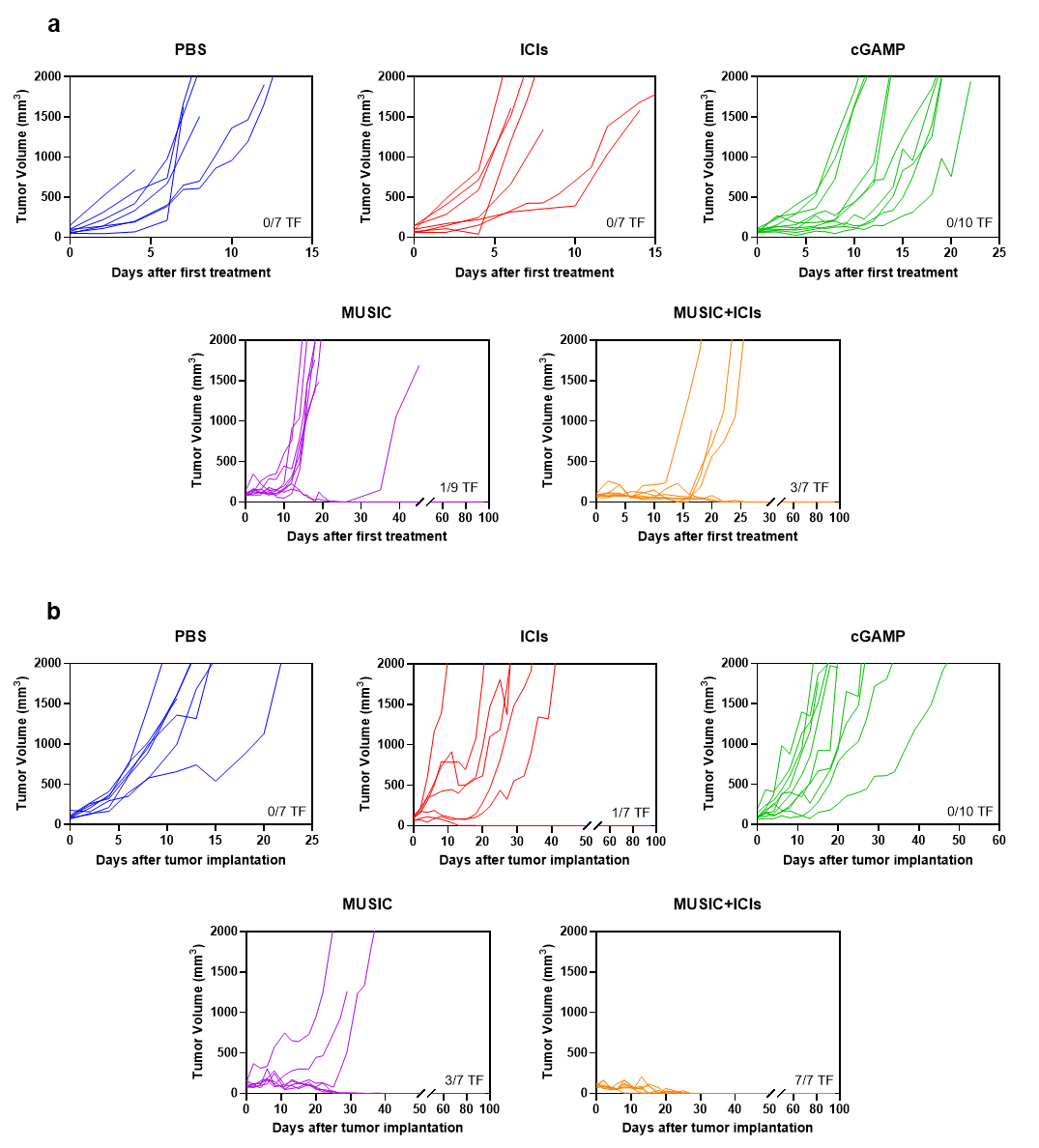


**Figure S3.** Spider plots of individual tumor growth curves. Tumor growth was monitored for 95 days after initial treatment or when tumors reached the euthanasia limit (volume over 2000 mm^3^ or 2 cm in any direction). **a**) B16F10. **b**) D4M.3A


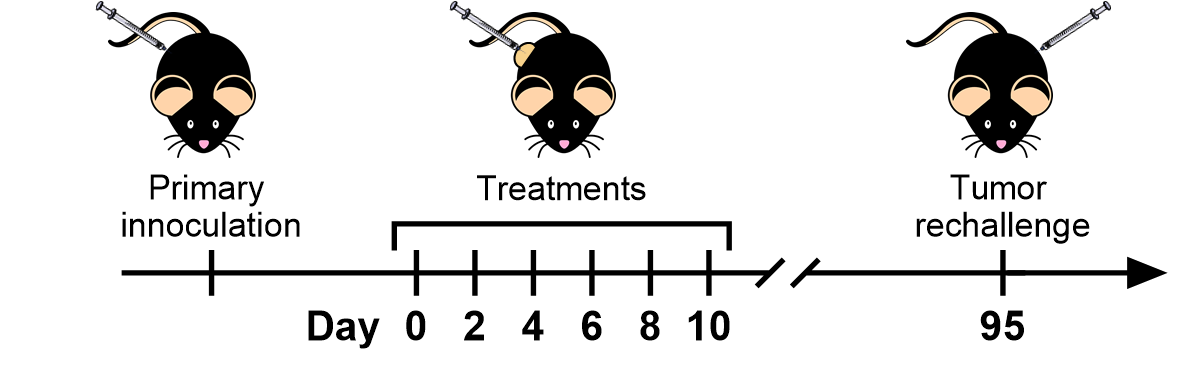


**Figure S4.** Rechallenging scheme. Mice that underwent complete tumor eradication after treatment were reimplanted with the same cell line on the contralateral flank 95 days after their first treatment.


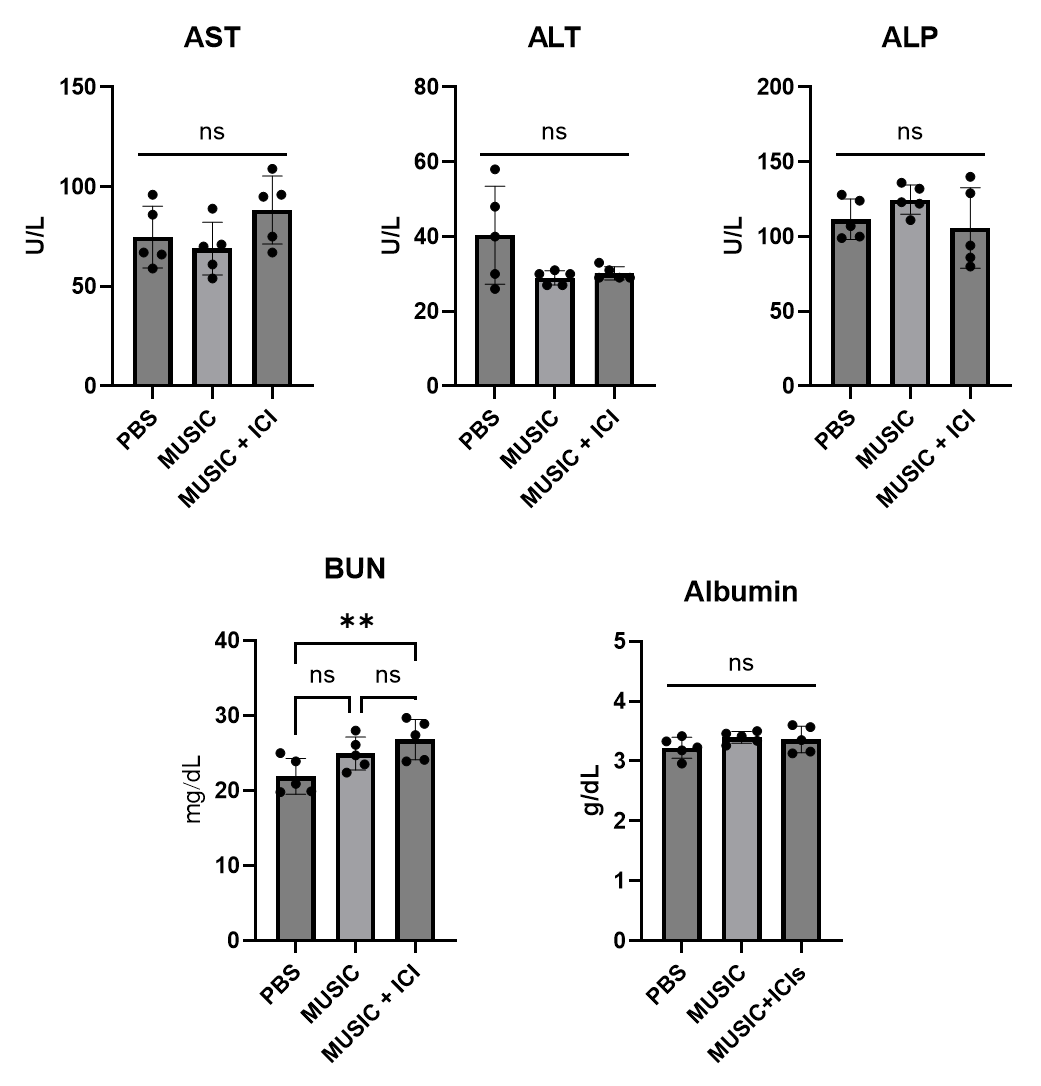


**Figure S5.** Evaluation of systemic toxicity after MUSIC treatment. Aspartate transferase (AST), alanine transferase (ALT), alkaline phosphatase (ALP), blood urea nitrogen (BUN), and albumin levels in mice were measured from blood samples 24 h after the indicated treatment regimens. *n =* 5 replicates for all groups. The data represent mean ± s.d. Data were analyzed by one-way ANOVA with Fisher’s LSD. p values > 0.05 were considered not significant (ns), p values < 0.05 were considered significant. **p value < 0.01.


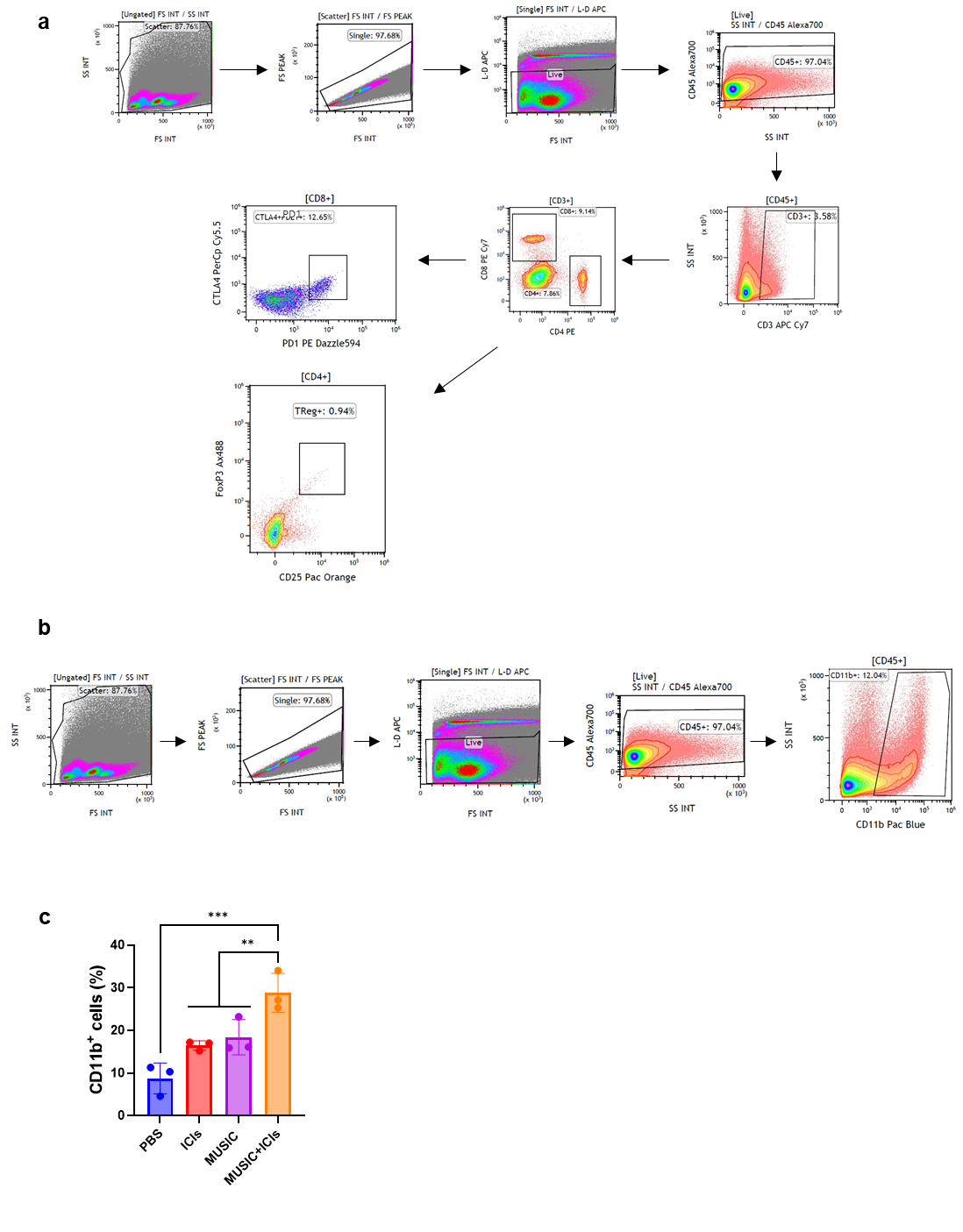


**Figure S6.** Flow cytometry gating strategy for analyzing immune cell populations. **a**) Flow cytometry gating strategy for experiments in **Figs. 3d-f and 4c-e**.

**b**) Flow cytometry gating strategy for analyzing myeloid populations to quantify the percentage of CD11b^+^ cells in D4M.3A tumor samples. **c**) Bar graph representation of the percentage of CD11b^+^ cells in tumor samples. Data are representative from three biologically independent experiments. Data are shown as mean ± s.d. and analyzed by one-way ANOVA with Fisher’s LSD. ***p* < 0.01, ^***^*p* < 0.001.
